# Distinct patterns of infiltrating CD8+ T cells in HPV+ and CD68 macrophages in HPV- oropharyngeal squamous cell carcinomas are associated with better clinical outcome but PD-L1 expression is not prognostic

**DOI:** 10.18632/oncotarget.14796

**Published:** 2017-01-22

**Authors:** Kenneth Oguejiofor, Henry Galletta-Williams, Simon J. Dovedi, Darren L. Roberts, Peter L. Stern, Catharine M.L. West

**Affiliations:** ^1^ Translational Radiobiology Group, Division of Molecular & Clinical Cancer Sciences, School of Medical Sciences, Faculty of Biology, Medicine and Health, University of Manchester, Manchester Academic Health Science Centre, Christie Hospital NHS Trust, Manchester, UK; ^2^ Targeted Therapy Group, Division of Molecular & Clinical Cancer Sciences, School of Medical Sciences, Faculty of Biology, Medicine and Health, University of Manchester, Manchester Academic Health Science Centre, Christie Hospital NHS Trust, Manchester, UK; ^3^ Immunology/Childrens Cancer Group, Division of Molecular & Clinical Cancer Sciences, School of Medical Sciences, Faculty of Biology, Medicine and Health, University of Manchester, Manchester Academic Health Science Centre, Christie Hospital NHS Trust, Manchester, UK

**Keywords:** checkpoint inhibitor, PD-L1, human papillomavirus, oropharyngeal squamous cell carcinomas, tumour infiltrating lymphocytes

## Abstract

Immunotherapies are beginning to revolutionise treatment paradigms in oncology with monoclonal antibodies (mAb) targeting T-cell co-inhibitory (e.g. PD-1/PD-L1) and co-stimulatory pathways (e.g. CTLA-4/CD28) demonstrating clinical utility. Some clinical studies demonstrate that responsiveness to PD-1/PD-L1 mAb therapy is greater in patients with expression of PD-L1 in the tumour microenvironment. However, robust responses have also been observed in patients with low or absent expression of PD-L1. Using multiplex immuno-fluorescent labelling we sought to determine how infiltration of tumours by CD8^+^ T-cells, their expression of PD-1, and the expression of PD-L1 on both tumours and CD68 cells (macrophages) correlated with HPV status and outcome in a cohort of 124 oropharyngeal squamous cell carcinomas (OPSCC).

## INTRODUCTION

The natural history of tumour development may be sculpted by the immune system with multiple mechanisms identified that facilitate immunological escape including down-regulation of HLA, altered composition of regulatory cells in the tumour microenvironment and up-regulation of suppressive immune checkpoint pathways [1, 2]. Recent clinical studies targeting co-inhibitory and co-stimulatory immune checkpoints such as cytotoxic T lymphocyte protein-4 (CTLA-4), programmed cell death 1 (PD-1) and its ligand (PD-L1) have demonstrated anti-tumour activity in several cancer types [3–6].

Chronic activation of T-cells leads to an exhausted phenotype which is typically identified in tumour-infiltrating T cells [7]. T-cell expression of PD-1 is indicative of this phenotype and signalling through its ligand, PD-L1, can attenuate signalling through the T-cell receptor (TCR) and lead to anergy/apoptosis and contribute to immune escape. The expression of PD-L1 by tumour infiltrating immune cells (including B and T lymphocytes, dendritic cells, and macrophages), vasculature, endothelium, malignant cells and in the associated stroma can provide for significant immune regulation [8–13].

Expression of PD-L1 is inducible in responses to changes in inflammatory mediators in the tumour microenvironment such as interferon (IFN)γ and tumour necrosis factor (TNF)α making PD-L1 a dynamic and potentially problematic biomarker. It is perhaps unsurprising that there is heterogeneity in responsiveness to PD-1/PD-L1 monoclonal antibody (mAb) therapy in relation to expression of PD-L1 in the tumour microenvironment [14]. Indeed, up-regulation of PD-L1 during treatment may be indicative of therapeutic response to checkpoint inhibitor treatment [15].

In oropharyngeal squamous cell carcinoma (OPSCC), better clinical outcomes are reported in HPV^+^ compared to negative patients irrespective of treatment and this is linked to differences in immune infiltrating T cells in the tumour and/or stromal compartments [16, 17]. Persistent infection is the key risk factor in the natural history of HPV associated cancers [18] and in the oropharynx this may be facilitated by the presence of “immune privileged” sites provided by expression of PD-L1 in tonsil crypts [19]. However, higher expression of PD-L1 in HPV^+^ compared negative OPSCC has been reported but levels were not correlated to improved patient survival in either group [20]. Another study showed that improved survival can also be associated with increased expression of PD-1^+^ T cells in HPV positive OPSCC [21].

The aim of this study was to investigate immune factors present in HPV^+^ and negative OPSCC at the time of diagnosis and in particular assess the potential of PD-L1 expression as a biomarker for either prognosis or treatment selection. The approach used was multiplex immuno-fluorescent labelling to quantitate infiltration of tumours by CD8^+^ T-cells, their expression of PD-1, and the expression of PD-L1 on both tumours and CD68 cells (macrophages) correlated with, HPV status and outcome in a cohort of 124 OPSCC.

## RESULTS

### Patient characteristics

Samples were available from 124 patients, whose tumours showed congruency of HPV^+^ or negative phenotype by three detection methods and had sufficient material for analysis. Of these 75 (60%) were positive for HPV and 49 (40%) were HPV negative. Patient characteristics of this subgroup were not significantly different from previous analyses published on 139 tumours [16] (*X*^2^ *P* = 0.56). Table 1 summarises patient characteristics stratified by HPV status showing significant differences (*X*^2^) in smoking status (*P* = 0.02), T stage (*P* = 0.0002) and N stage (*P* = 0.01). There were significantly more smokers with HPV^-^ tumours (*P* = 0.02), and significantly more HPV^+^ tumours were larger (*P* = 0.0002) and had nodal disease (*P* = 0.01) at diagnosis.

**Table 1:** Clinicopathological characteristics stratified by HPV status

Characteristic		HPV-ve %	HPV+ve %	*P*
**Age**	**median**	60	58.5	0.44
**Alcohol**	**Never** **Low** **High**	3 88 9	7 76 17	0.08
**Smoking**	**Never** **Ex** **Current**	18 45 37	35 45 25	0.02
**T stage**	**1** **2** **3** **4**	8 37 21 34	23 41 25 11	0.0002
**N stage**	**0** **1** **2** **3**	27 14 59 0	25 21 51 3	0.01
**AJCC staging**	**I** **II** **III** **IV**	2 12 15 71	2 13 23 62	0.5
**Grade**	**Well** **Mod** **poorly**	6 61 33	4 49 47	0.12
**PD-L1 expression**	**<5%** **≥5%**	74 26	84 16	0.8

### CD8 T cell infiltration

Using multiplex fluorescence, there were higher CD8^+^ T cell densities in HPV^+^ compared to negative OPSCC in both tumour and stromal areas (MWU; *P* = 0.03, *P* = 0.02). These data reproduce our previous results using chromogenic detection [16]. There were no significant differences in the densities of CD8^+^PD-1^+^ T cells between HPV^+^ and negative OPSCC in either tumour or stromal sites (Table 2a). However, there were slightly more CD8^+^PD-1^+^ as a percentage of total CD8^+^ T cells in HPV negative (23%) than positive (19%) tumours in stroma and/or tumour areas (Table 2a). This might be important as CD8^+^ T cell stromal densities link best to improved outcome.

**Table 2:** The mean cell density or expression of different T cell populations

Immune cell type	HPV status	All	Tumour	Stroma
Table 2a – Median cell density of T cells per ROI (3.5 × 10^5^ mm^2^)				
**CD8+**	**HPV positive**	14.7	9	3.6
	**HPV negative**	9.6	6.2	1.6
	***P***	*0.01*	*0.03*	*0.02*
**CD8+PD-1+**	**HPV positive**	2.8	1.5	1.2
	**HPV negative**	2.2	1.3	1
	***P***	*0.1*	*0.5*	*0.8*
Table 2b The mean percentage expression of PD-L1				
**PD-L1+**	**HPV positive**	3.1	2.3	0.5
	**HPV negative**	6.1	3.2	2.1
	***P***	*0.01*	*0.20*	*0.01*
Table 2c Median cell density of macrophages per ROI (3.5 × 10^5^ mm^2^)				
**CD68**	**HPV positive**	46	34	8
	**HPV negative**	35	25	7
	***P***	*0.08*	*0.01*	*0.1*
**CD68+PD-L1+**	**HPV positive**	3.1	1.4	1.2
	**HPV negative**	5.6	1.9	3
	***P***	*0.004*	*0.4*	*0.005*

### PD-L1 expression in OPSCC

OPSCC PD-L1 labelling expressed as a mean percentage of cells (±SEM) was 4.22±1.0%. The proportion of patients with PD-L1 overall tumour positivity greater than 5%, irrespective of HPV status, was 21% (42/200), with 16% (16/100) and 26% (26/100) for HPV^+^ and negative tumours respectively (Table 1). There was significantly lower mean overall PD-L1 expression in HPV^+^ (3.1±1%) compared to negative (6.1±2%) tumours (MWU, *P* = 0.01) (Table 2b). Stratifying PD-L1 expression by site of expression, (stroma versus tumour areas) showed a higher PD-L1 expression in the tumour regions. However HPV^+^ tumours had lower stromal PD-L1 expression when compared with negative tumours (MWU, *P* = 0.01; Table 2b). The data indicate that the higher PD-L1 expression in HPV^-^ tumours results from increased stromal expression of PD-L1. This pattern of expression is consistent with potential interference of the function of CD8^+^PD-1^+^ T-cells in the stroma. One source of PD-L1 expression might be infiltrating macrophages and this was investigated by analysing CD68^+^PD-L1^+^ expression.

### CD68 infiltration and PD-L1 expression

There were more CD68 positive cells in the tumour area of HPV^+^ compared to negative OPSCC (MWU, *P* = 0.01) and a non-significant increase in the stromal regions (Table 2c). Overall, 7% of the CD68 cells expressed PD-L1 in HPV^+^ compared with 16% in negative OPSCC (Table 2c). Interestingly, CD68^+^PD-L1^+^ stromal densities were also significantly lower in HPV^+^ compared to negative OPSCC (MWU, *P* = 0.005). This is consistent with the greater expression of PD-L1 in HPV¯ compared to HPV^+^ OPSCC (Table 2b) being due to PD-L1 expression on CD68 cells in the stroma. Supplementary Figure 2 illustrates staining of HPV^+^ and negative tumours showing observable higher infiltration in the tumour and stroma of the HPV^+^ tumour.

Our previous studies showed that for HPV^+^ tumour patients, a higher density of CD8^+^ T cells in the stroma was associated with overall better outcome. However, within this group, it is possible that the effect of relatively high CD8^+^ T cell infiltration could be modulated due to PD-1 activation on the T cells and its interaction with the PD-L1 ligand expressed by either CD68 or tumour cells in some patients. By contrast, HPV¯ OPSCC have lower CD8^+^ T cell but higher densities of CD8^+^ PD-1^+^ T cells and CD68^+^PD-L1^+^ macrophages in their stroma compared to HPV^+^ tumours. This differing balance of immune infiltration in HPV^-^ tumours might contribute to the overall poorer clinical outcome of these patients compared to those with HPV^+^ tumours.

### Immune factors and clinical outcome

Kaplan-Meier analysis of overall survival (OS) or local regional control (LRC) of all patients stratified by levels above or below the median for CD8^+^, CD8^+^PD-1^+^ T cells, CD68^+^, CD68^+^PD-L1^+^ cells or total PD-L1^+^ populations showed no significant associations (Table 3).

**Table 3:** Univariate analysis of immune cell markers in all patients

Markers	LRC (HR, 95% CI)	*P* value	OS (HR, 95% CI)	*P* value
**PD-L1**	0.67, 0.34–3.50	0.78	1.21, 0.51–2.80	0.65
**CD8+**	0.45, 0.54–4.50	0.87	1.03, 0.49–2.13	0.93
**CD68+**	1.40, 0.24–2.30	0.65	1.24, 0.59–2.60	0.55
**CD68+PD-L1+**	0.56, 0.34–2.60	0.89	0.68, 0.32–1.41	0.30
**CD8+PD-1+**	1.10, 0.45–3.40	0.57	0.72, 0.34–1.53	0.40

**Figure 1:**
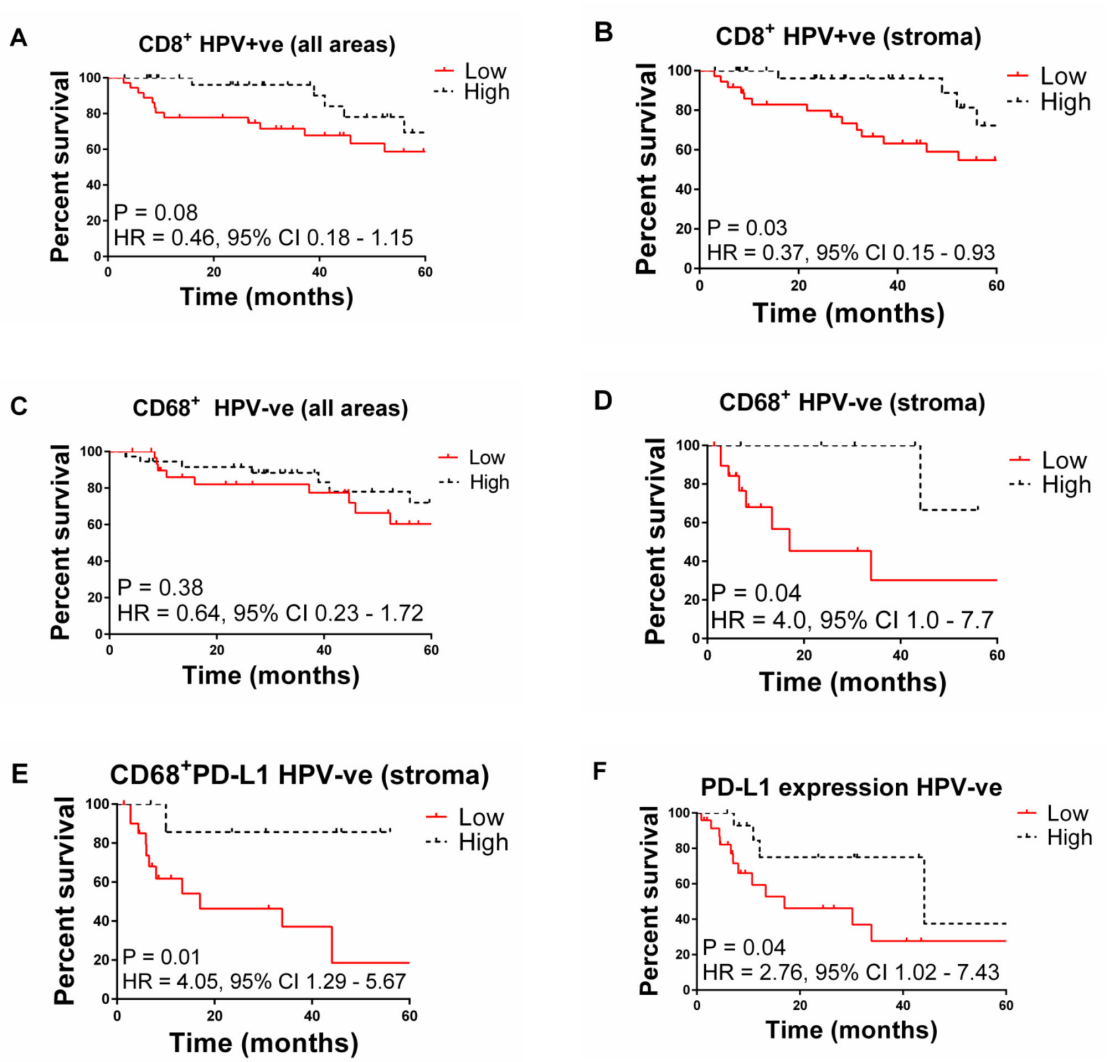
Kaplan-Meier plots of overall survival for OPSCC patients stratified by **A**. high vs. low CD8^+^ T cell infiltration in HPV^+^ tumours (all areas), **B**. high vs. low CD8^+^ T cells in HPV^+^ tumours (stroma), **C**. high vs. low CD68^+^ cell infiltration in HPV^+^ tumours (all areas), **D**. high vs. low CD68^+^ T cells in stroma of HPV^-^ patients (stroma), **E**. high vs. low CD68^+^PDL1 cells in HPV^-^ tumours (stroma), **F**. high vs. low PDL1^+^ cell infiltration in HPV^-^ patients.

However, stratification of patients by HPV status reveals that in HPV^+^ OPSCC patients, CD8^+^ density in the stroma correlated with improved survival. There was no association for the CD68^+^ and CD68^+^PD-L1^+^ levels in the stroma to associate with a better outcome (Table 4; Figure 1). The majority of PD-L1 is expressed by the tumour cells in the HPV^+^ OPSCC. Interestingly, higher expression of PD-L1 in tumour cells showed a trend for a worse outcome (*P* = 0.06). There was no correlation between PD-L1 tumour expression and CD8^+^ T cell density in the stroma or tumour areas. For CD68 infiltration there was a positive correlation with CD8^+^ T cell levels in tumour (*r* = 0.40; *P* = 0.01) but not stroma (*r* = 0.15, *P* = 0.34).

**Table 4:** Univariate analysis of immune cell markers in tumour and stroma areas in HPV positive and negative patients

Markers	Patients	LRC (HR, 95% CI)	*P* value	OS (HR, 95% CI)	*P* value
**HPV+ve** **Tumour**	CD8+ CD8+PD-1+ CD68+ CD68+PD-L1+ PD-L1	1.56, 0.45–3.40	0.34	1.25, 0.60–2.61	0.55
		0.90, 0.60–2.10	0.56	1.00, 0.50–1.89	0.34
		0.90, 0.45–2.30	0.76	1.14, 0.37–3.44	0.81
		0.70, 0.12–1.50	0.67	0.78, 0.67–2.34	0.56
		0.45, 0.23–1.25	0.08	0.34, 0.10–1.07	0.06
**HPV+ve** **Stroma**	CD8+ CD8+PD-1+ CD68+ CD68+PD-L1+ PD-L1	0.25, 0.12–1.05	**0.05**	0.37, 0.15–0.93	**0.03**
		0.89, 0.67–1.40	0.15	0.35, 0.10–1.20	0.12
		1.30, 0.50–3.20	0.23	4.0, 1.0 – 7.7	0.07
		1.20, 0.34–2.30	0.56	1.30, 0.23–2.00	0.45
		1.45, 0.60–3.40	0.76	1.62, 0.40–6.00	0.46
**HPV–ve** **Tumour**	CD8+ CD8+PD-1+ CD68+ CD68+PD-L1+ PD-L1+	0.90, 0.20–3.40	0.78	0.56, 0.13–2.30	0.56
		0.80, 0.34–5.60	0.59	0.45, 0.10–3.40	0.45
		0.56, 0.23–1.70	0.56	0.94, 0.23–3.75	0.93
		2.50, 0.90–3.40	0.08	3.10, 0.82–5.70	0.09
		0.45, 0.12–0.78	0.78	0.38, 0.11–0.65	0.88
**HPV–ve** **Stroma**	CD8+ CD8+PD-1+ CD68+ CD68+PD-L1+ PD-L1+	0.56, 0.12–3.60	0.67	0.67, 0.23–4.50	0.68
		0.78, 0.34–2.70	0.78	0.45, 0.15–3.70	0.57
		3.20, 0.95–5.70	**0.05**	4.00, 1.00–7.70	**0.04**
		3.60, 1.50–4.70	**0.03**	4.00, 1.29–5.67	**0.01**
		3.50, 1.80–6.70	**0.04**	4.60, 1.23–7.00	**0.02**

By contrast in the HPV¯ OPSCC patients, infiltration of CD68 cells (*P* = 0.04) especially CD68^+^PD-L1^+^ cells (*P* = 0.01) and increased expression of PD-L1 in the stroma was associated with significantly improved survival (Figure 1D-1F). One might speculate that immune control of HPV¯ tumours includes a greater role for macrophage activity compared to HPV^+^ tumours. Most PD-L1 expression was associated with CD68 cells as the levels were significantly correlated.

### Immune cell characteristics associated with improved survival

**Figure 2:**
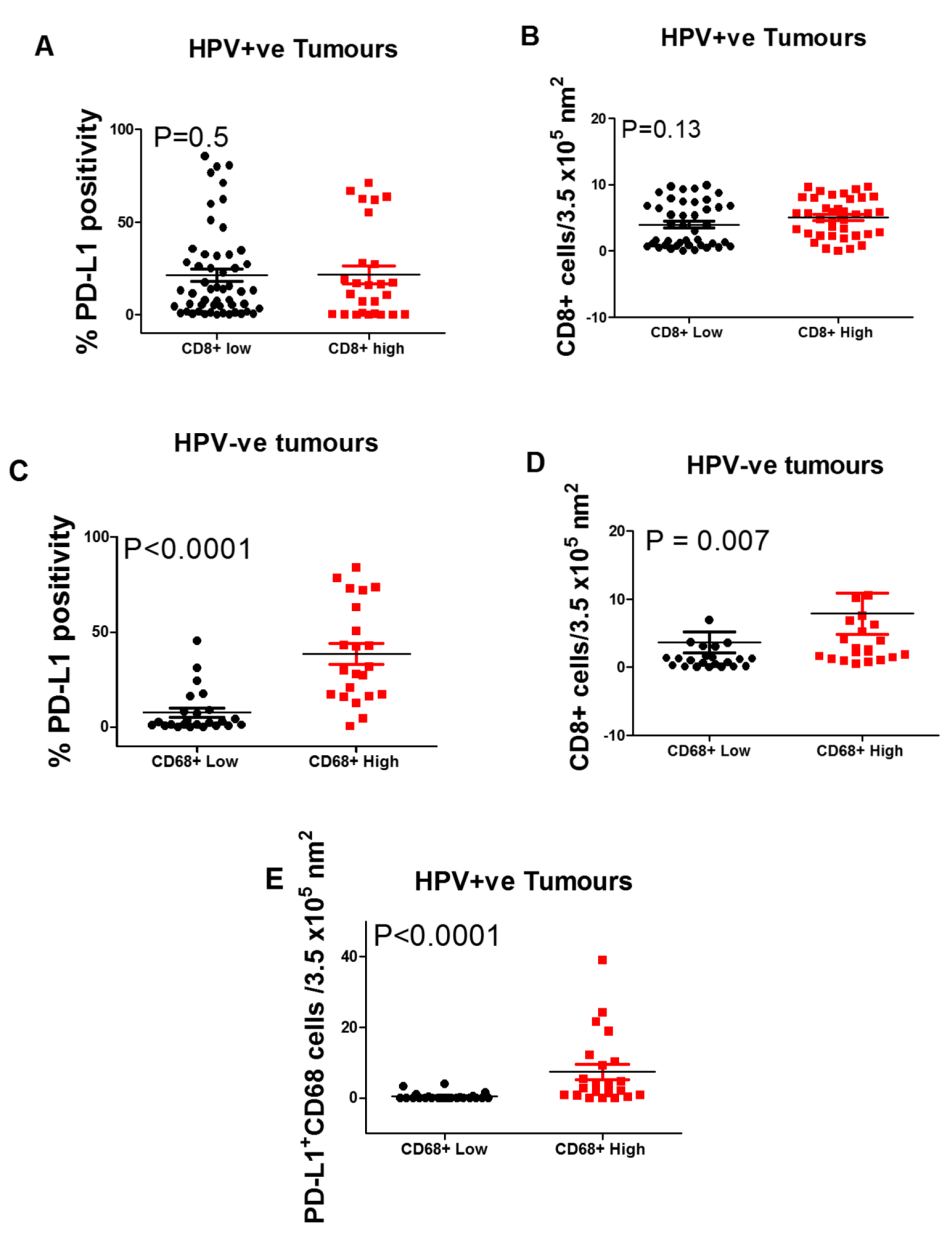
Box Plots showing the percentage of PD-L1+ **A**. and CD68+ **B**. cells in HPV positive oropharyngeal squamous cell carcinoma with low vs. high CD8+ T cell densities. Also, the expression of PD-L1 **C**. and densities of CD8+ **D**. and CD68+PD-L1+ cells in HPV negative tumours with a low vs. high density of CD68+ cells. Stratified by (A) PD-L1 expression and B) CD68+ cell density. **C**., **D**. and **E**. show significant differences in high vs. low CD68+ cell stratified by PD-L1 expression (*P* < 0.0001), CD8+ density (*P* = 0.007), and CD68+PD-L1+ (*P* < 0.0001) densities respectively.

Stratifying HPV^+^ patients by median CD8^+^ T cell density showed that there was no statistically significant difference in either PD-L1 expression or CD68 cell infiltration (Figure 2A & 2B). For HPV¯ tumours sub-stratifying by median CD68 cell density into high versus low groups showed a significantly higher level of PD-L1^+^ expression (*P* < 0.0001), (Figure 2C) and density of both CD8^+^ T cells (*P* = 0.007) and CD68^+^PD-L1^+^ cells (*P* < 0.0001) (Figure 2D & 2E). In summary, for HPV^+^ patients, PD-L1 expression does not correlate with clinical outcome but in the HPV¯ tumour group, higher PD-L1 expression by CD68 cells is associated with a relatively better survival.

## DISCUSSION

In this study, immune CD8^+^ T and CD68^+^PD-L1^+^ cell densities differed between HPV^+^ positive and negative OPSCC and were associated with differing outcomes. This may reflect a different natural history of oncogenesis with divergent immune control mechanisms apparent in the cancers at time of diagnosis. Furthermore, these differences pre-treatment might predict and influence the prevailing mechanisms post treatment.

It is now well established that tumour infiltration by immune cells can impact disease outcome in several cancer types [22]. Higher densities of CD8^+^ T cells in tumour and stroma compartments of HPV^+^ OPSCC and the relationship with patient survival has been described in this cohort [16] and replicated in this study. The potential mechanism likely involves an active immune response (evident by increased stromal infiltration of CD8^+^ T cells) which can be recruited following therapy. This model has been observed in pre/post treatment biopsies in melanoma where a stepwise accumulation of CD8^+^ T cells initially at the invasive margin and centre of the tumour was described in patients responding to immune modulating agents [15]. In contrast, there was no difference in CD8^+^PD-1^+^ T cell infiltration between HPV^+^ and negative patients and no relationship with patient outcomes. Interestingly there was a higher percentage of CD8^+^ T cells expressing PD-1 in the stroma compared with tumour regions. Others have made similar observations, with significant differences in CD8^+^ T cell density or CD8^+^PD-1^+^ T cell infiltration between HPV^+^ and negative OPSCC tumours [21]. However, Lyford-Pike et al described no difference between the level of PD-1 expression on CD8^+^ T cells in the OPSCC and that seen on CD8^+^ T cells around inflamed tonsils [19]. This suggests induction and activation of PD-1 on CD8^+^ T cells in response to inflammation. Although PD-1 is expressed after ligation of the T cell receptor [23], there are no data to support the function of PD-1 in the absence of signalling via its ligand (PD-L1) [24]. PD-L2, another co-receptor for PD-1, can also be an inhibitory molecule; it is expressed by antigen-presenting cells, other immune and non-immune cells in an inducible manner mainly through Th2-associated cytokines. However, it is not clear that PD-L2 function has any relevance to the tumour microenvironment [25].

It appears that the density of CD8^+^PD-1^+^ T cells alone is insufficient to comment on functionality. In this study, our observation of almost equal density of CD8^+^PD-1^+^ in HPV^+^ or negative tumours but higher expression of PD-L1 in HPV^-^ tumours suggests possible greater inhibition of CD8^+^ T cell function in HPV^-^ tumour patients. This might contribute to the relative improved prognosis in HPV^+^ patients. A recent study [26] showed higher numbers of IFN-γ and IL17^+^ CD8^+^ T cells, myeloid dendritic cells and pro-inflammatory cytokines as well as higher mRNA for PD-1 in HPV^+^ compared to negative head and neck squamous carcinomas. This later study used cancers from beyond the OPSCC subgroup and the analysis used disaggregated tumour tissue and flow cytometry but confirmed the predominance of CD8^+^ T cells in HPV associated tumours but without any tissue/ tumour context.

There have been several studies looking at PD-L1 expression in head and neck SCC (HNSCC) or OPSCC but interpretation and comparison is complicated because of differences in pathological criteria, HPV typing, antibody reagents, immuno-histochemical methodologies and scoring criteria [20]. The experience for the use of PD-L1 as a prognostic marker in cancers has been mixed [24]; PD-L1 tumour expression has correlated with poorer outcome in breast [27] and renal [28, 29] but better survival in lung cancer [30].

In this study, we used the approach described by Herbts et al in evaluating predictive correlates to anti-PD-L1 antibody treatment. This demonstrated that a 5% positivity threshold for infiltrating cells but not tumour labelling correlated with non-small cell lung carcinoma patient (NSCLC) response to therapy [31]. In the latter study, 101 HNSCC (without HPV status) were also labelled using the same methodology and identified 27% as positive with respect immune infiltrating cells and 19% with tumour cell associated labelling, but there were insufficient patients for relative outcome to therapy assessment. In our study, the percentage of patients with PD-L1 overall specimen positivity greater than 5%, irrespective of HPV status was 21%, with 26% and 16% for HPV- and positive OPSCC respectively. The use of the cut-off at 5% is obviously arbitrary and was used here as a result of the positive association with response to checkpoint inhibitor treatment in NSCLC patients [31]. It is clear that differences in PD-L1 expression by different cancer types, immune or tumour cell populations and their distribution within the tumour could all potentially influence the clinical outcome. There are many different antibodies and methodologies in use for PD-L1 labelling and as yet no properly validated approach useful for prognostication. Contextualisation of tumour and or immune cell expression of PD-L1 with immune cell infiltrate and a validated methodology provides more information about the relevant immune micro-environment. Thus, in our study the majority of PD-L1 was expressed by the tumour cells in the HPV^+^ OPSCC but with no correlation with CD8^+^ T cell density in the stroma or tumour areas. There was a non-significant trend for PD-L1 expression on tumour cells to be an adverse prognostic factor in patients with HPV^+^ OPSCC, which may reflect a role in the inhibition of cytotoxic T cell function in some of these patients. This suggestion is supported by a previous study in oral SCC which reported a lower density of infiltrating CD8^+^ T cells with higher PD-L1 tumour expression, although this was not linked to patient survival or stratified by HPV status [32].

We also found that the infiltrating immune cells which were PD-L1^+^ were mostly CD68^+^ cells; 16% of CD68 cells expressed PD-L1 in HPV¯ compared to 7% in the HPV^+^ OPSCC. This putative macrophage infiltration had no significant association with patient survival in HPV^+^ patients. However, in HPV¯ patients increased infiltration of CD68 cells (particularly PD-L1+) into the stromal compartment was associated with improved patient survival. This contradicts the suggestion that CD68 macrophage expression of PD-L1 serves as a homeostatic mechanism which inadvertently provides an immune privileged milieu encouraging tumour growth [19]. However, CD68^+^PD-L1^+^ cell presence could reflect a feedback mechanism to restrict T cell responses through PD-L1 attenuation of CD8^+^ T cells and thus might reflect a beneficial local anti-tumour response. However if this is the case there were insufficient effector T cells to quantitate in HPV¯ tumours.

This study did not distinguish between the differentiation states of the macrophage e.g. pro-inflammatory (M1) or suppressive (M2) macrophage phenotypes. These phenotypes represent a simplification of the dynamic functional plasticity of these cells which can differentiate according to paracrine, autocrine and cell surface stimuli. With this rider, “M1 and M2 macrophages” are recruited to sites of inflammation with the overall effect resulting from the balance of types. In HPV¯OPSCC one might speculate a predominant infiltration of M1 macrophages known to exert an anti-tumour effect [33]. This theory is further strengthened by: 1) the observation of significantly higher cytotoxic T cell densities and 2) increased expression of PD-L1 in patients with higher macrophage densities. Increased PD-L1 expression may be related to increased secretion of IFN-γ by cytotoxic T cells and M1 type macrophages in these patients which is known to increase PD-L1 expression [34].

It is suggested that in cancer TIL responses may be attenuated by the proximity to immune inhibitory factors such as PD-L1 with expression on tumour cells promoting T cell apoptosis in in-vitro and in-vivo models [35, 36]. Importantly, both intrinsic and extrinsic factors have been linked to PD-L1 expression on tumour cells. Intrinsic oncogenic changes upregulating PD-L1 include increased proliferation potential [27]. increased signalling via signal transducer and activator of transcription 3 (STAT3), extracellular regulated kinases (ERK) and epidermal growth factor receptor (EGFR) [37–40], inactivation of phosphatase and tensin homolog (PTEN) [41] and the ALK mutation in lung cancer [42]. Up-regulation of PD-L1 by extrinsic factors has been shown from immune cell signalling via pathogen recognition receptors such as Toll like receptor (TLR), and by IFN-γ [43–45]. Increased PD-L1 expression in a background of increased immune effector cell density might define patients with an anti PD-1/PD-L1 targetable phenotype. Thus PD-L1 expression, type and density of immune cells must be co-evaluated.

Pre-treatment immune phenotypes as described above may predict response to therapy but the therapy may also influence the differing effector mechanisms. Radiotherapy is commonly used in the treatment of OPSCC and was the consistent feature of all the patients studied here and its abscopal effects may be central to immune activity and/or recruitment post treatment [46]. This could include a role for immunogenic tumour cell death (following radiotherapy) leading to efficacious antigen presentation and T cell stimulation, reduction in local Treg activity and subsequent repopulation/modification of the tumour microenvironment [47–50]. The success of the treatment protocols may be dependent on “a critical threshold” of CD8^+^ T cells and/or absence of a highly suppressive immune micro-environment [51]. In this study, HPV^+^ patients with higher CD8^+^ T cells in pre-treatment biopsies might predict patients responding with a favourable immunogenic profile post radiation. Similarly, in HPV^-^ patients with putatively less immunogenic tumours, increased recruitment of macrophages expressing PD-L1 reflects a favourable immune milieu that can be stimulated following radiotherapy and/or augmented with immunotherapy.

In summary, we describe differing local immune profiles in HPV^+^ and negative OPSCC which can relate to better clinical responses. It is important to acknowledge that tumours derived from different parts of the oropharynx may not all have the same natural history in respect of HPV neoplasia and immune surveillance [52]. Future studies should examine these factors in the different oropharynx sites of the tonsils, base of tongue, lateral walls and the soft palate. Sufficiently powered studies following patient tumour immune phenotypes pre- and post-radiotherapy are required to investigate treatment immune relationships. Clearly there is no simple pattern of PD-L1 expression that can be used as a biomarker for either prognosis or treatment selection in OPSCC at this time.

## MATERIALS AND METHODS

### Patient selection and HPV detection

The study design was retrospective. An audit using a radiotherapy database at The Christie NHS Foundation Trust Hospital identified patients with a confirmed histological diagnosis of OPSCC. Patients were treated between January 2002 and December 2011 with radiotherapy as one or the only therapy modality. Details of the treatment can be found elsewhere [53]. Patients treated with a palliative intent were excluded. Four treatment options were available to patients in this study: radiotherapy alone, radiotherapy in combination with chemotherapy and/ or surgery. 50% of the patients received radiotherapy alone, 22% received radiotherapy post-surgery, 17% received radiotherapy in combination with chemotherapy and 11% had surgery in combination with chemoradiotherapy. Patient clinico-pathologic and outcome data were collected from the case notes and the Christie head and neck assessment forms. The study was approved by the National Health Service (NHS) Health Research Authority (HRA) National Research Ethics Service (NRES) committee in the North West (reference number 03/TG/076). Individual patient consent was not required. Pre-treatment formalin-fixed paraffin-embedded (FFPE) blocks prepared at biopsy were requested. To limit impact of the close association of lymphoid and epithelial tissue in tonsillar and base of tongue origin OPSCC, all sections included in the study were prepared from blocks with clearly distinct tumour tissue. The possibility of inclusion of bystander immune cells resulting from the anatomic location is reduced and any influence likely to be insignificant.

As described in [16] patients with concordant HPV positivity or negativity using three methods were included in the study. The three methods were p16 immunohistochemistry, HPV DNA PCR and HPV DNA ISH. Patients with discordant HPV results were not included in the study.

### Multiplex tumour infiltrating lymphocyte immunohistochemistry

FFPE sections 4 µm thick were stained using the Ventana auto staining platform (Ventana Medical Systems, Tucson, Arizona, US). The semi-closed system was used for de-paraffinisation, epitope retrieval, endogenous peroxidase blockade and secondary antibody detection. The multiplex protocol used involved an initial high pH (8.5) and subsequent low pH (6.0) in between fluorescent labeling. The use of the tyramide-signal amplification (TSA) based system (Opal multiplex TSA system [PerkinElmer, Waltham, Massachusetts, US]) for fluorescent staining allowed for repeated staining steps. 100 μl of antibody and opal detection reagent were hand applied. The first antibody in the staining algorithm was a rabbit monoclonal antibody (mAb) against PD-L1 (Cell Signaling, Danvers, Massachusetts, US; 1:200) detected using opal cyanine 5.5. An intervening antigen retrieval step followed each detection step. The second antibody was a mouse anti-CD8 mAb (clone C8/144B; Dako, Glostrup, Denmark; 1:60) detected using opal cyanine 3. Third and fourth antigen staining steps were mouse mAbs against PD-1 and CD68 (Abcam, Cambridge, UK; 1:50 and 1:200) detected by fluorescein and cyanine 3.5 respectively. The slides were then removed from the Ventana, submerged for 3 × 5 minutes in EZ preparation to remove the oil film, cover-slipped with Prolong aqueous mounting agent and counterstained with DAPI (Cell Signalling).

### Multiplex IHC automated image analysis, scoring and data analysis

For each slide, the Vectra automated multispectral imaging system (PerkinElmer, Massachusetts, USA) was used to perform both low (x4) and high (x20) power scans of 30 randomly selected tissue grids. Spectral libraries generated from single stained slides using the Nuance FX multispectral imaging system software (PerkinElmer, Massachusetts, USA) were then loaded into inForm advanced image analysis software (PerkinElmer). Manual segmentation of tissue and stromal regions was done on selected tissue grids. The individual biopsy TIL density per region of interest (ROI; 3.5 x10^5^ nm) was determined from 30 randomly selected ROIs of tumour or stromal areas for each section. The median T-cell density for each group was used to stratify patients into “high” or “low” TIL groups. PD-L1 labeled specimens were scored as positive at a threshold of 5% of all stained cells as previously described; this approach in a study of 20 OPSCC patients had predicted those subsequently responded to anti-PD-L1 therapy [31]. In addition, the individual contributions of PD-L1 expression by tumour, T cell and CD68 cell populations were determined. Supplementary Figure 1 shows examples of staining: actual image (A); a composite bright field image (B); the delineation of tumour (red) and stroma (green) compartments (C); and image A de-convoluted to show the different marked populations resolved from their distinct spectra in the image (D-G).

### Statistical analyses

Image analysis data were exported to Microsoft Excel worksheets. Charts and data comparisons were performed using SPSS version 20.0 and GraphPad Prism. For the Box Plots shown, Shapiro-Wilk normality test was used to test for the distribution of the data. As the data were mostly not normally distributed, the Mann-Whitney U (MWU) test for comparing the data was used. Actuarial calculations of locoregional control (LRC) and overall survival (OS) were obtained using the Kaplan-Meier method. Univariate analysis was compared using the Log Rank (Mantel-Cox) method. Chi-squared test was used to compare categorical data and the threshold for statistical significance was 0.05. As the data showed non-parametric statistics Spearman rank correlation coefficient was used to assess the relationship between variables.

## SUPPLEMENTARY MATERIALS FIGURES AND TABLES


