# Supplementary material for: Distinct patterns of infiltrating CD8+ T cells in HPV+ and CD68 macrophages in HPV- oropharyngeal squamous cell carcinomas are associated with better clinical outcome but PD-L1 expression is not prognostic
Source: Oncotarget. 2017 Jan 22;8(9):14416–27. doi: 10.18632/oncotarget.14796 (PMC5362415; doi:10.18632/oncotarget.14796)
Supplement: Supplementary file 1 [file oncotarget-08-14416-s001.pdf]

# Distinct patterns of infiltrating CD8+ T cells in HPV+ and CD68 macrophages in HPV- oropharyngeal squamous cell carcinomas are associated with better clinical outcome but PD-L1 expression is not prognostic

## Supplementary Information

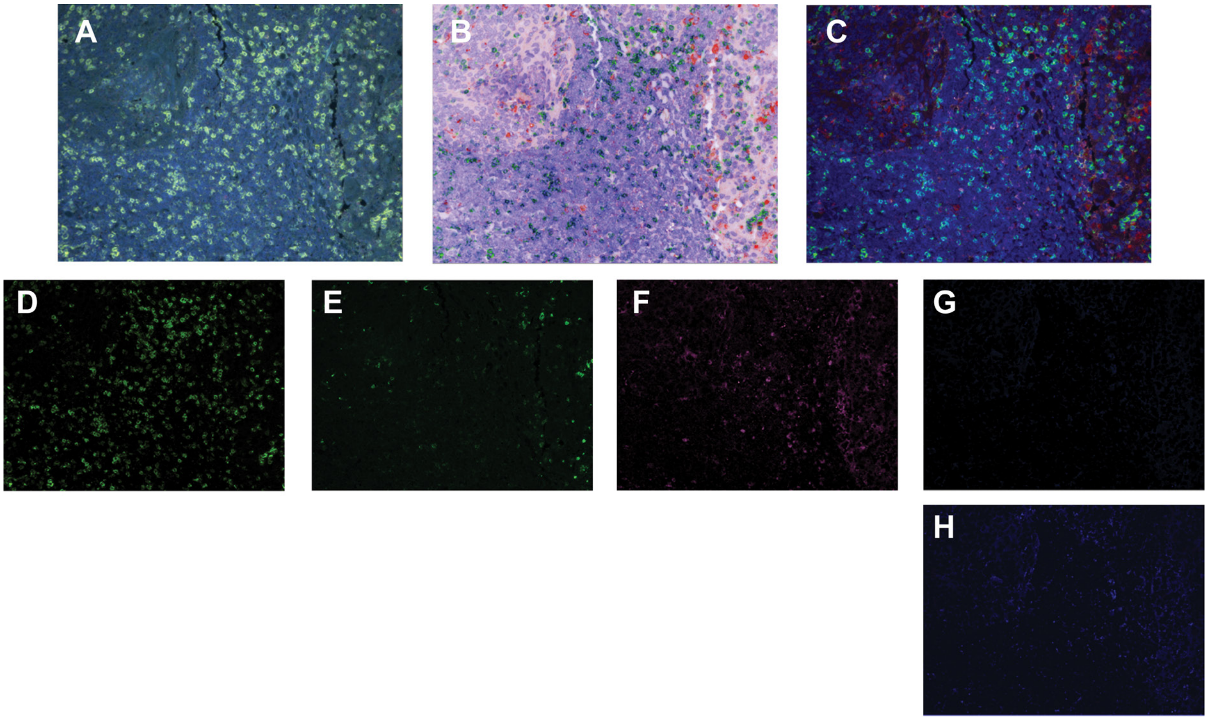


**Supplementary Figure 1.** Example of fluorescent stained multiplex images for marker expression. A) actual image, B) composite image (bright field), C) composite image (fluorescent) with red (CD8+), green (CD68+), pink (PD-L1+) and magenta (PD1+) colours, separated into tumour (red) and stroma (green) compartments. De-convoluted of image (A) shows individual spectrums for CD68+ (D), CD8+ (E) PD-L1 (F) and PD1 (G). PD1 spectra has been rescaled for clarity (H) but was not used in the analysis.

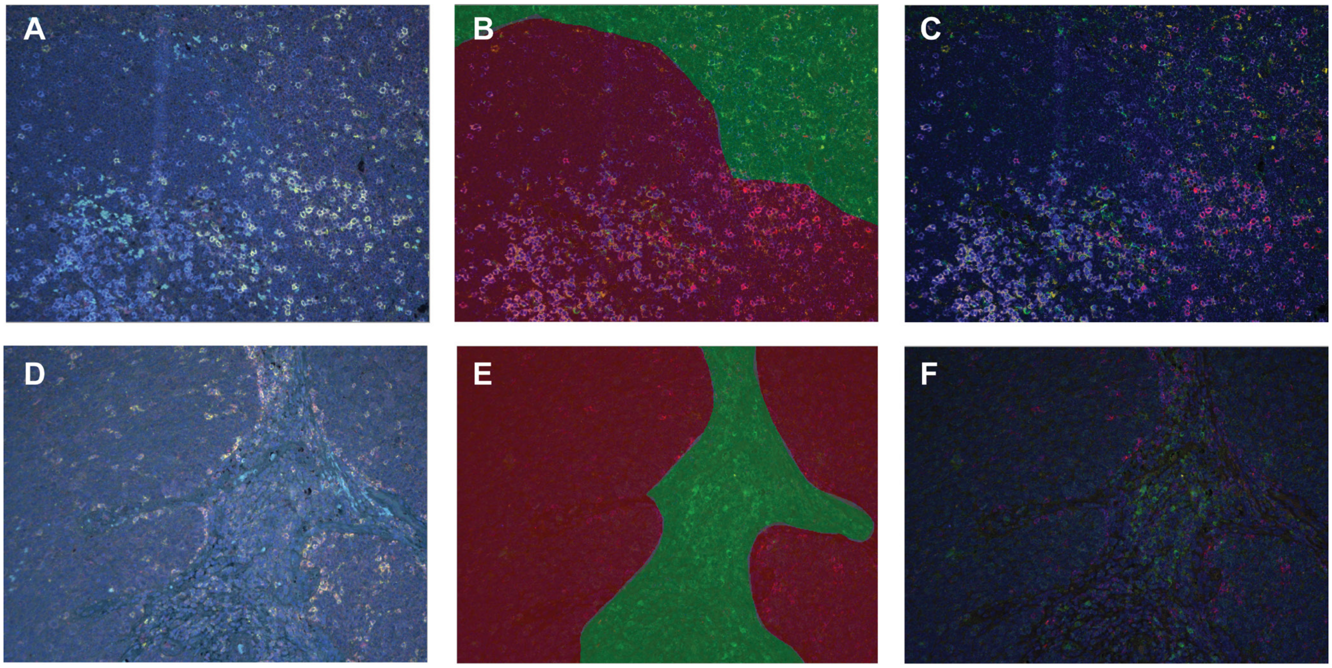


**Supplementary Figure 2.** (A) Actual example staining of an HPV positive tumour segmented into (B) tumour (red) and stroma (green). Also, (C) the composite image showing yellow (CD8+), pink (CD8+PD1+), red (CD68+), magenta (CD68+PD-L1+) and green (PD-L1+) stained cells. (D) Actual example staining of an HPV negative tumour segmented into, (E) (red) and stroma (green) and (F) composite image. The composite image shows yellow (CD8+), pink (CD8+PD1+), red (CD68+), magenta (CD68+PD-L1+) and green (PD-L1+) cells.
